# Multi- and Transgenerational Effects of Environmental Toxicants on Mammalian Reproduction

**DOI:** 10.3390/cells11193163

**Published:** 2022-10-09

**Authors:** Paola Rebuzzini, Gemma Fabozzi, Danilo Cimadomo, Filippo Maria Ubaldi, Laura Rienzi, Maurizio Zuccotti, Silvia Garagna

**Affiliations:** 1 Laboratory of Developmental Biology, Department of Biology and Biotechnology “Lazzaro Spallanzani”, Via Ferrata 9, University of Pavia, 27100 Pavia, Italy; 2 Clinica Valle Giulia, GeneraLife IVF, Via De Notaris 2B, 00197 Rome, Italy; 3 Department of Biomolecular Sciences, University of Urbino “Carlo Bo”, Via Sant’Andrea 34, 61029 Urbino, Italy; 4 Centre for Health Technologies (CHT), University of Pavia, Via Ferrata 5, 27100 Pavia, Italy

**Keywords:** environmental toxicants, endocrine-disrupting compounds, fertility, reproduction, multigenerational effect, transgenerational effect, folliculogenesis, spermatogenesis, oocyte, sperm, epigenetic inheritance

## Abstract

Environmental toxicants (ETs) are an exogenous chemical group diffused in the environment that contaminate food, water, air and soil, and through the food chain, they bioaccumulate into the organisms. In mammals, the exposure to ETs can affect both male and female fertility and their reproductive health through complex alterations that impact both gametogeneses, among other processes. In humans, direct exposure to ETs concurs to the declining of fertility, and its transmission across generations has been recently proposed. However, multi- and transgenerational inheritances of ET reprotoxicity have only been demonstrated in animals. Here, we review recent studies performed on laboratory model animals investigating the effects of ETs, such as BPA, phthalates, pesticides and persistent contaminants, on the reproductive system transmitted through generations. This includes multigenerational effects, where exposure to the compounds cannot be excluded, and transgenerational effects in unexposed animals. Additionally, we report on epigenetic mechanisms, such as DNA methylation, histone tails and noncoding RNAs, which may play a mechanistic role in a nongenetic transmission of environmental information exposure through the germline across generations.

## 1. Introduction

Since the beginning of the industrial revolution, a plethora of synthetic compounds has been synthetized and used for many industrial and agricultural activities. Their progressive exponential employment and consequent waste determine their diffusion and accumulation into the environment. They are highly and widely diffused in the environment, contaminate water, air and soil [1] and, through the food chain, bioaccumulate into organisms. Only at the beginning of the 1990s, the detrimental effects and the toxicity of these compounds emerged worldwide, and their impact on human and animal health was first reported during the Wingspread Conference in 1991.

Exposure to environmental toxicants (ETs) induces reprotoxic effects by altering the production, maturation and quality of gametes and the reproductive cycle in both males and females and the delivery and pregnancy outcomes and may determine premature reproductive senescence (aging) [2].

During the past 30 years, declining human fertility has become a global public health priority. Infertility affects nearly one in six couples worldwide (about 15% of couples in the reproductive age), with a progressive increasing incidence [3,4]. The causes of male and female infertility are very heterogeneous [3], and the exposure to ETs concurs with its incidence [5,6,7]. Interestingly, recent studies suggested that ETs are also key players in shaping future health outcomes [8] not only for those who are directly exposed to them but also for upcoming generations. However, the inheritance of infertility from parents to offspring has been clearly demonstrated in model animals but only proposed in a few recent papers for humans [9,10]. In our species, transmission across generations has been reported for some disorders, such as asthma, obesity and cardiovascular diseases [11,12].

ETs include several categories of compounds, very heterogeneous in their chemical structure and mechanism of action. For example, ETs, such as butadiene, chloroprene, isoprene, acephate, cypermethrin and cadmium, are genotoxic compounds able to induce genetic mutations, DNA breaks and/or chromosomal rearrangements or affect the enzymes involved in DNA replication (inheritable damage) [13,14]. Others, which represent the most abundant group, do not constitute a well-defined category at the structural and functional levels, and their detrimental effects on reproduction may be exerted through mechanisms that include interference with the endocrine system. Among ETs, the endocrine-disrupting compounds (EDCs) encompass almost 800 different chemicals [15], including both natural and synthetic compounds, categorized into three major groups (i.e., pesticides, chemicals in consumer products and in food–contact materials) [16]. They alter the activation, synthesis, secretion and binding of endogenous physiological hormones, thus affecting several hormonal and metabolic processes [17,18,19]. More specifically, they act by mimicking hormones (mainly estrogens and/or androgens), binding to their receptors and promoting inappropriate responses at improper times or by directly blocking their action [1,20,21]. EDCs were initially thought to exert their actions primarily through nuclear hormone receptors, i.e., estrogen, androgen, progesterone, thyroid and retinoid receptors. Subsequent studies highlighted that they also act via non-nuclear steroid hormone receptors (e.g., membrane estrogen receptors); non-steroid receptors (e.g., serotonin, norepinephrine or dopamine receptors); orphan receptors (e.g., aryl hydrocarbon receptors); enzymatic pathways involved in steroid biosynthesis and/or metabolism and numerous other mechanisms, all converging upon the endocrine and reproductive systems [21]. Some EDCs have genotoxic effects, causing the hydroxylation of deoxyguanosine and/or DNA strand breaks (both single and double), thereby promoting malignant transformation of the affected cells [22,23,24].

Recently, it has been reported that ETs perturb epigenetic marks, such as DNA methylation and histone modifications. The epigenetic programming laid down during development can be modified by exposure to ETs, affecting sensitive periods, such as fetal and/or perinatal phases, and causing immediate adverse outcomes [1,25,26]. Additionally, alterations of the methylation profiles and acetylation landscapes occurring during fetal and/or perinatal life may predispose individuals to the development of pathologies that will emerge years later in childhood or adult life [27,28,29] and, more dramatically, that may even be transmitted across generations [30,31,32,33].

## 2. Environmental Toxicants Exposure and Reproductive Health

In mammals, the exposure to high concentrations of ETs can affect both male and female fertility and their reproductive health. In both sexes, ETs may alter the hypothalamus–pituitary gland–gonads axis [34,35] by acting as agonists or antagonists of hormonal receptors (e.g., GnRH, FSH and LH receptors), impairing their expression or their signal transduction processes (for details, see [36]). Additionally, ETs interfere with the endogenous GnRH, FSH, LH, testosterone, estradiol and progesterone synthesis, transport, distribution and/or clearance [21,36].

Both in vivo and in vitro experiments have demonstrated that, in adult females, direct exposure to several ETs causes interference or disruption of the normal steroidogenesis in the ovary by affecting numerous steroidogenic enzymes (e.g., steroidogenic acute regulatory protein and Cyp family) and hormone levels (e.g., progesterone) [37,38,39,40,41,42]. ETs increase the risk for ovarian and uterus cancer, birth defects in newborns and the spontaneous loss of pregnancy [43]. Several ETs, targeting the ovary, adversely affect folliculogenesis and follicle/oocyte health [44]. ET exposure has been associated with decreased antral follicle counts and reduction of the oocytes number [45,46,47,48,49], inhibition of antral follicle growth by increasing the expression of the proapoptotic factor and the induction of follicle atresia [42,50,51,52]. Additionally, ET exposure causes a decreased oocyte quality [53,54,55] and reduced or blocked in vivo ovulation in rodents [56].

When females are exposed to ETs during the perinatal period or prepubertal phase, the latter a physiological period characterized by the development of secondary sexual characteristics, gonadal maturation and attainment of their reproductive capacity [57], the female reproductive system can be even more severely impaired than when exposed during adulthood [58,59,60]. In fact, prepuberty exposure to a single or a mixture of ETs alters ovary functionality, proper follicles recruitment and contributes to earlier pubertal onset, accelerating or anticipating the processing of maturation of secondary sexual characteristics [36].

In adult males, direct exposure to ETs, such as BPA or phthalates, significantly impairs testosterone production [61,62,63,64,65] and reduces the expression level of androgen receptors (AR) and spermatogenesis-related genes by disrupting the enzymes involved in hormonal production (e.g., CYP450aom, 3β-HSD, 17β- HSD, ODF1 and TNP1 [66,67]). Additionally, acting as antiandrogens or mimicking estrogens, ETs have dramatic consequences on the production of healthy sperm [18,68]. In general, reduced testicular weight [68], abnormal sperm count (necrozoospermia and/or oligozoospermia), lower motility (asthenozoospermia), sperm morphology alterations, genome damage, disomy of chromosomes X and Y and hyperhaploidy are the main defects reported in ET-exposed adult males [69,70,71,72,73,74]. Moreover, ET exposure reduces the proliferation of spermatogenic cells; activates apoptotic signaling pathways (intrinsic apoptosis pathway: activation of caspases 3 and 9, Bax and cytochrome C); suppresses antiapoptotic factors (*Bcl-2*) [75,76,77,78,79] and alters sperm LINE-1 hydroxymethylation [80] and DNA hydroxymethylation [81].

Overall, following ET exposure, adult males are less affected than adult females, likely because of the presence of spermatogonial stem cells (that represent about 0.03% of all germ cells) in the testes warrants the continuous production of sperm, thus attenuating negative repercussions on male fertility [82]. On the contrary, the fixed, nonrenewable pool of germ cells in the ovaries determines the major susceptibility of females to this category of compounds.

## 3. ETs Exposure Effects Transmitted across Generations

**Figure 1:**
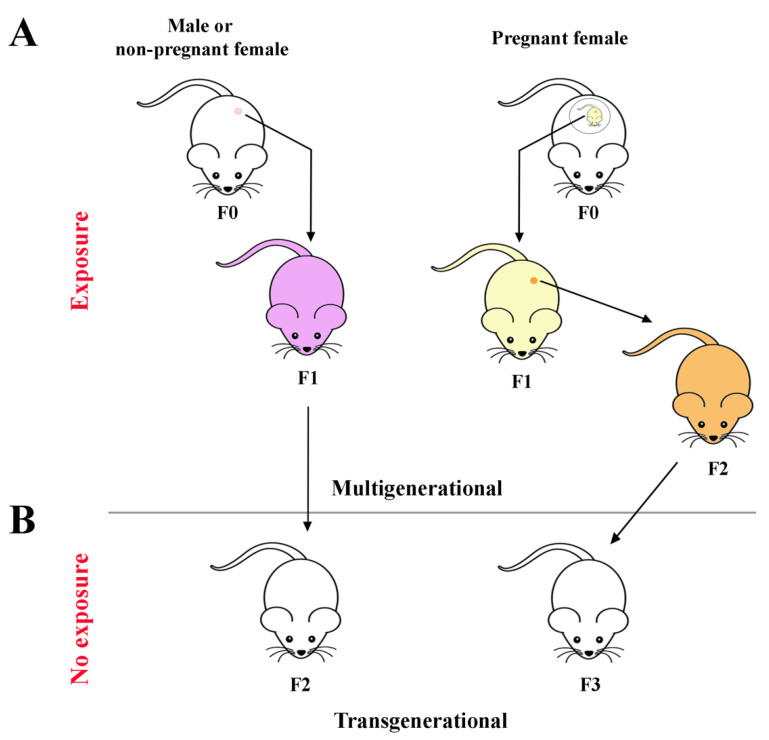
Schematic representation of the (**A**) multigenerational and (**B**) transgenerational transmission of ETs effects. F0: parental generation; F1: first filial generation: F2: second filial generation; F3: third filial generation.

The ET effects described above refer to individuals who have been directly exposed in their postnatal, prepubertal or adult life. In vitro, in vivo and epidemiological studies have reported that several ETs can induce effects that are transmitted through generations [33,83,84,85]. Direct exposure during adulthood generates alterations that may be passed to children and then to grandchildren through epigenetic changes that occurred in the parents’ germline. Preconception exposure of F0 (ancestral) adult males or nonpregnant female germ cells gives birth to a F1 litter, which may be affected (multigenerational transmission) (Figure 1A). Although not directly exposed, the F2 generation may present ET-induced effects (transgenerational transmission) (Figure 1B) [86].

Due to their biochemical features, several ETs are able to pass the placental barrier and reach the developing fetus, causing a direct in utero fetal exposure, with doses comparable to that of the mother [87]. The developing fetuses (F1) of exposed pregnant F0 females are themselves considered directly exposed, as is the F2 generation (Figure 1A). The subsequent F3 generation is the first that is not directly exposed to the compound(s), but any inherited ET-induced effects are due to a “transgenerational” transmission [86] (Figure 1B).

The transmission across generations of ET-induced effects on both male and female reproduction has been reported for both synthetic and natural compounds.

In this review, we focus on phytoestrogens and mycotoxins within the category of natural compounds and on BPA, phthalates, pesticides and persistent contaminants, as they represent the most diffused and dangerous anthropic compounds spread nowadays in the environment. We report on studies performed using laboratory model species, the mouse and the rat, to highlight the ET-induced multi- and transgenerational reprotoxic effects on both males and females. Then, the epigenetic mechanisms that play a pivotal role in passing environmental information through gametes in mammals are discussed.

### 3.1. Phytoestrogens and Mycotoxins

Only a handful of papers describes the alterations induced by phytoestrogens [88,89] and mycotoxins [90,91,92].

Exposure to 40 mg/kg/day genistein during gestation (2 weeks before delivery) and after weaning significantly increased testes weights, diameters of the seminiferous tubule and heights of the seminiferous epithelium of offspring mice, together with increased testosterone levels. Exposure to a very high dose, i.e., 800 mg/kg/day, significantly decreased testes weight and sizes. Additionally, it increased the mRNA expression of *ESR2* (*p* < 0.05), *CYP19A1*, *SOX9* and *BRD7* and decreased the expressions of *SOX9* and *BRD7*. Additionally, at the same dose of exposure, a higher ratio of apoptotic germ cells and abnormal spermatozoa was detected [88] (Table 1). A longer exposure to a lower genistein dose (1 mg/kg) from E1 to PND21 decreased the expression of *Daz*, a gene encoding an RNA-binding protein important for spermatogenesis, and of *Stra8*, *Spo11* and *Sycp3*, genes involved in the regulation of the meiotic initiation of spermatogenesis. Additionally, the expression of *Star*, *Cyp11a1* and *Cyp17a1*, genes crucial for the steroidogenesis pathway, significantly dropped [89] (Table 1).

Ancestral mycotoxin Zearalenone (ZEN) exposure induced, in the F1 generation of females, a significant increase in the follicle-stimulating hormone concentration, a decrease of estradiol, follicular atresia and a thin uterine layer, together with a reduced expression of estrogen receptor-alpha and of gonadotropin-releasing hormone receptor [90]. In F1 male mice, prenatal ZEA exposure diminished spermatozoa motility and concentration, decreased the 5-methylcytosine (5-mC) level, increased H3K9 and H3K27 histone methylation markers and reduced estrogen receptor-alpha [91]. Additionally, ZEA generated abnormal vacuole structures and loose connections in the testes and decreased the testosterone levels and semen quality [92] (Table 1).

**Table 1:** Phytoestrogens and Mycotoxins-Induced Effects In Model Animals Across Generations.

Species	Sex	Exposure	Dose	Effects across Generations	Reference
Mouse	Male	Genistein Two weeks before delivery. After weaning up to PND 35.	40 mg/kg BW */day 800 mg/kg BW/day	F1: increased serum testosterone levels (40 mg/kg); increased testis weight (40 mg/kg); decreased testis weight (800 mg/kg); higher diameter of seminiferous tubules (40 mg/kg); increased heights of seminiferous epithelium (40 mg/kg day); smaller diameter of seminiferous tubules (800 mg/kg); increased *ESR2, CYP19A1* (all doses), *SOX9* and *BRD7* (40 mg/kg) mRNA expression in the testis; decreased *SOX9* and *BRD7* mRNA expression (800 mg/kg) in the testis; increased number of apoptotic germ cells (800 mg/kg); abnormal sperm (800 mg/kg)	[88]
Rat	Male	Genistein from E1 to PND 21	1 mg/kg BW/day	F1: increased expression of *Daz* in the testis F2: increased expression of *Stra8, Spo11* and *Sycp3*; decreased expression of *Fas* in the testis; decreased expression of *Star*, *Cyp11a1*, *Cyp17a1* in the testis	[89]
Rat	Female	Zearalenone From E0 to 21	5, 10, and 20 mg/kg	F1: increase of follicle-stimulating hormone concentration (10 and 20 mg/kg); estradiol decrease (10 and 20 mg/kg); follicular atresia (20 mg/kg); thin uterine layer (20 mg/kg); reduced expression of estrogen receptor-alpha (10 and 20 mg/kg) in the placenta; reduced expression of gonadotropin-releasing hormone receptor (10 and 20 mg/kg) in the placenta	[90]
Mouse	Male	Zearalenone From E12.5 to E18.5	20 µg/kg BW/day 40 µg/kg BW/day	F1: decreased sperm motility; decreased sperm concentration (all doses); reduced testis weight (all doses); percentage alteration in the of cells at different stages of meiosis (increased percentage of leptotene cells; decreased percentage of diplotene cells; all doses); reduction of 5hmC (all doses) in the testis; increased percentage of H3K27me3-positive spermatogonial cells (all doses); increased expression of H3K9 in the testis; increased expression of G9a in the testis; reduced percentage of ERα-positive Leydig cells (all doses)	[91]
Mouse	Male	Zearalenone From E1 a E18	2.5 and 5.0 mg/kg BW/day	F1: abnormal vacuole structures; testes loose connections (all doses); decreased semen quality (all doses); decreased sperm count (all doses); decreased testosterone levels (all doses)	[92]

* BW: body weight.

### 3.2. Bisphenol A

BPA is a widely diffused plasticizer employed in the manufacturing process of polycarbonate plastics, epoxy and vinyl ester resins, PVC, polyurethane and thermal paper. Its potent estrogenic endocrine disruptive activity has been widely investigated, and for its estrogen-mimetic action, it affects the reproduction of exposed adult males and females [93]. Several recent papers have reported its impact also across generations, and some significant examples of these effects are hereafter reported.

Ancestral exposure to increasing BPA doses during fetal gonadal sex determination (from embryonic days (E) 7 to E14) generated F1 mouse male offspring that developed testes with reduced weight, stage-dependent modification of the lumen area and germ cells apoptosis, but these alterations were not detected in the F2 and F3 offspring generations [94] (Table 2). However, a more recent, thorough study of the seminiferous epithelium organization demonstrated a significantly augmented number of seminiferous tubules with the loss of germ cells, decreased the frequency of seminiferous epithelium stage VIII and dislodged the testicular cellular organization in both F1 and F2 males (Table 2) [95]. The same study showed, at the highest dose tested (Table 2), a higher percentage of 5-mC/total DNA, indicating an altered DNA methylation level in F1–F2 spermatozoa. Additionally, a >two2-fold induced expression of PHGPX, ATP5O, GSTM5, NDUFA10 and ASRGL1 proteins was revealed in both the F1 and F2 generations, whereas the differential expression of SOD2 was noticed only in F1. In the F3 progeny, the sperm proteome expression profile returned to the unexposed control levels, whereas a higher percentage of 5-mC was still present [95] (Table 2). Overall, these results indicate that BPA has detrimental effects on the spermatogenetic process of directly exposed animals, whereas, in the not directly exposed F3 generation, spermatogenesis was recovered.

Differently from the mouse, in the F3 male rat generation, exposure to BPA during the same developmental window resulted in testes dysfunctions and in 197 differential DNA methylation regions (DMRs) in gene promoters in sperm [96,97], highlighting the presence of both cellular and epigenetic alterations in a generation that has not been directly exposed to BPA. Continuous ancestral exposure, during the whole fetal period, to low BPA doses induced changes in the lipid metabolism, protein secondary structures and testosterone production in Leydig cells in the F1 and F2 generations [98]. In particular, when both parents were exposed to BPA, the F0 and F1 generations showed a decline of testosterone levels due to structural and functional alterations of the Leydig cells, including mitochondrial damage and endoplasmic reticulum stress [98] (Table 2). However, only the BPA-induced decrease in testosterone production was transmitted to the F2 generation.

In rat females, ancestral exposure to BPA during fetal gonadal sex determination (from E8 to E14) induced pubertal abnormalities and ovarian diseases, mainly in F3 animals [96,97] (Table 2). In the mice, BPA exposure for a longer fetal period (E11 to birth) inhibited ovarian germ cell nest breakdown and reduced litter sizes only in F1 (Table 1) [99], suggesting that these BPA-induced alterations were not carried over into subsequent generations. Interestingly, a reduction of ovarian follicle numbers, delayed puberty, altered estrous cyclicity, decreased conception rates and fertility were observed up to the F3 generation. In the same animals, the altered gene expression of steroidogenic enzymes was observed from the F1 to the F3 generation (Table 2) [52,99,100], suggesting the multi- and transgenerational transmission of all these effects.

**Table 2:** BPA-Induced Effects In Model Animals Across Generations.

Species	Sex	Exposure	Dose	Effects across Generations	Reference
Mouse	Male	From E7 to E14	50 μg, 5 mg, and 50 mg/kg BW */day	F1: reduced testis weight (50 mg/kg at PND ^+^ 30; 5 and 50 mg/kg at PND 60); alterations in seminiferous epithelial stages (all doses at both PND 30 and PND 60) (increased lumen area of stage VII; decreased lumen area of stage VIII); apoptosis of germ cells (5 and 50 mg/kg); F2: drop of stemness properties of spermatogonia (5 mg/kg)	[94]
Mouse	Male	From E7 to E14	50 μg, 5 mg, and 50 mg/kg BW/day	F1: decreased frequency of stage VIII testicular seminiferous epithelial cells (5 and 50 mg/kg); increased number of abnormal seminiferous tubules (5 and 50 mg/kg); decreased sperm count (5 and 50 mg/kg); decreased sperm motility (50 mg/kg); altered DNA methylation in spermatozoa (5 and 50 mg/kg); proteomic expression changes in spermatozoa (50 mg/kg) F2: decreased frequency of stage VIII testicular seminiferous epithelial cells (50 mg/kg); increased number of abnormal seminiferous tubules (50 mg/kg); disruption of testicular germ cell organization (50 mg/kg); disruption of spermatogenesis (5 and 50 mg/kg); decreased sperm count (50 mg/kg); decreased sperm motility (50 mg/kg); altered DNA methylation in spermatozoa (50 mg/kg); proteomic expression changes in spermatozoa (50 mg/kg) F3: altered DNA methylation in spermatozoa (50 mg/kg)	[95]
Rat	Male	From E8 to 14	Mixture: BPA 50 mg/kg DEHP 750 mg/kg DBP 66 mg/kg BW/day	F3: pubertal abnormalities; testis dysfunction; apoptosis of spermatogenic cell; differential DNA methylated regions in spermatozoa	[97]
Rat	Male	Continuous during the whole fetal life	0.5 mg/kg BW/day	F0 and F1: changes in lipid metabolism in the testis; altered protein secondary structures in the testis; decreased testosterone production F2: decline of testosterone level; structural and functional alterations of Leydig cells	[98]
Rat	Female	From E8 to 14	50 mg/kg BW/day	F3: pubertal abnormalities; primary ovarian insufficiency; polycystic ovaries	[97]
Mouse	Female	From E11 to birth	0.5, 20, 50 µg/kg BW/day	F1: inhibited ovarian germ cell nest breakdown (all doses); decreased fertility (all doses); reduced litter size (50 μg/kg); reduction of primordial follicles number and increase of primary follicles (0.5 and 50 µg/kg); increase of preantral follicles (high doses); altered estradiol levels (20 µg/kg); increased expression of steroidogenic enzymes and steroidogenesis-related genes (*Hsd17b1* and *Cyp14a1;* 50 µg/kg) in the ovary F2: preterm delivery; decrease of primary follicle (0.5 µg/kg); increase of preantral follicle (0.5 and 20 µg/kg); decrease of primordial follicles (20 and 50 µg/kg); increased expression of sex steroid hormone receptors (*Ers1* and *Ar*; 50 μg/kg); increased expression of steroidogenic enzymes and steroidogenesis-related genes (*Hsd17b1* and *Fshr* at 0.5 µg/kg; *Fshr*, *Cyp17a1*, *Hsd17b1*, and *Star* at 20 µg/kg) in the ovary F3: delayed puberty; altered estrous cyclicity (50 μg/kg); decreased fertility (0.5 μg/kg); decreased expression of sex steroid hormone receptors (*Ers1*; 0.5 μg/kg); increased expression of steroidogenic enzymes and steroidogenesis-related genes (*Fshr* and *Cyp17a1* at 50 µg/kg) in the ovary	[52,99,100]

^+^ PND: postnatal day; * BW: body weight.

### 3.3. Phthalates

Phthalates are widely used in building materials, food storage containers, personal care products and medical devices; they are also detected in indoor air and household dust [101,102]. Low-molecular-weight phthalates include diethyl phthalate (DEP), dibutyl phthalate (DBP) and diisobutyl phthalate (DiBP), whereas high-molecular-weight phthalates include di(2-ethylhexyl) phthalate (DEHP), benzyl butyl phthalate (BzBP) and di-isononyl phthalate (DiNP). Their impact on both male and female reproductive health is clearly demonstrated (for a recent review, [102]), and examples of multi- or transgenerational effects on mouse and rat animal models are hereafter reported (Table 3).

In utero exposure to DEHP during fetal gonadal sex determination (E7-E14) results in a reduced sperm number and motility from F1 to F4, disrupted spermatogonial stem cell colonization and altered proliferation and germ cell association/organization. Interestingly, following the transplantation of F3-derived spermatogonia, the testis germ cell disorganization phenotype was observed in the recipient unexposed mice, suggesting DEHP-induced alterations as an inherent property of spermatogonial stem cell [103]. Continuous fetal exposure to DEHP from E11 to birth reduced the serum testosterone level, altered the blood testis barrier integrity and impaired spermatogenesis, compromising mouse F3 male fertility. At the molecular level, a variation of 98.08% on the expression pattern of Y-chromosome genes was detected. Specifically, the *Sry* gene expression was downregulated, whereas that of *Eif2s3y*, *Cdyl* and *Zfy2* (genes known to be determinants for spermatogenesis) were upregulated [104]. Similarly, prenatal exposure to DBP, another diffused phthalate compound, impairs male reproduction in the F1 to F3 generations by altering spermatogenesis, the Sertoli cell number and inducing testis metabolic changes [105] (Table 3). Interestingly, exposure to both DEHP and DBP generates similar male fertility impairments that, in most cases, are transmitted up to the F3 generation.

As for males, exposure to DEHP also displayed multi- and transgenerational effects on female fertility [106,107,108,109]. Adult female mice exposed to DEHP showed reduced ovarian expression of estrogen receptor 1 (Esr1) in the F0, F1 and F2 generations [110], with a progressive decreased expression across generations. The exposure of lactating rat dams induced a decrease in the estradiol, testosterone and progesterone levels in their serum. Additionally, interestingly, their F1 progeny presented altered expressions of luteinizing hormone, follicle-stimulating, androgen, estrogen, progesterone and peroxisome proliferator-activated receptors and of 3β hydroxysteroid dehydrogenase, aromatase and steroidogenic acute regulatory proteins, indicating that the exposure of pups through lactation during postnatal life can also have dramatic consequences on the hormones involved in fertility regulation [111] (Table 3).

When DEHP exposure occurred in utero, an increase in the presence of fluid-filled ovarian cysts was observed only in F1 adult mouse ovaries, whereas impaired estrous cyclicity and a reduced total follicle number was inherited up to F3. A similar phenotype was observed in the F2 generation even at low DEHP doses, indicating a dose-independent response of several EDCs also in females [107]. Hormone production was also impaired [107], and in particular, the 17β-estradiol levels increased in the F1 and F2 generations [85] (Table 3). In ovaries of PND21 mice, DNA methyltransferase (*Dnmt*) expression and the presence of a 5-mC increment were detected in F1, and a reduction of *Tet* expression was observed in F2 and F3 animals, whereas a reduction of *Dnmt* and 5-mC expression was detected in the F3 only [112] (Table 3).

Alterations of hormone production were also obtained in F1 after the administration of a phthalate mixture [106,108,109], together with cystic ovaries and fertility complications in the F2 and F3 generations [106]. When the exposure was extended throughout the gestational and up to the end of the weaning period, PND21, an acceleration of follicle recruitment, decrease of the oocyte quality, embryonic developmental competence and modification of the expression profile of a panel of ovarian and preimplantation embryonic genes were observed up to the F3 generation [113] (Table 3).

**Table 3:** Phthalate-induced effects in model animals across generations.

Species	Sex	Exposure	Dose	Effects across Generations	Reference
Mouse	Male	DEHP from E7 to E14	500 mg/kg	F1–F4: disruption of testicular germ cell association; reduced sperm motility F3: alteration of spermatogonial stem cell function	[103]
Mouse	Male	DEHP from E11 until birth	20, 200 μg/kg/day	F3: decreased fertility (20 µg/kg); reduced testicular steroidogenic capacity (20 and 200 μg/kg); impaired spermatogenesis (20 and 200 μg/kg); decreased sperm concentration (20 and 200 μg/kg); decreased sperm motility (20 and 200 μg/kg); alteration of BTB integrity (20 and 200 μg/kg); alteration of Y genes expression (20 and 200 μg/kg) in the testis	[104]
Rat	Male	DBP from E8 to E14	500 mg/kg	F1–F3: spermatogenesis failure; altered reproduction; decrease sperm count; reduced Sertoli cells number; metabolic changes in the testis (increase level of betaine; drop of betaine homocysteine S-methyltransferase); DNA hypomethylation (in TM-4 cells, an immortalized cell line derived from mouse testis).	[105]
Mouse	Female	DEHP Adult (12 weeks old)	80 mg/kg/day	F0–F2: reduced expression of Esr1 in the ovary	[110]
Rat	Female	DEHP from PN day 1 to 21	1, 10, and 100 mg/kg/BW *	F0: decrease of estradiol (all doses), testosterone and progesterone levels (10 and 100 mg/kg) F1: altered mRNA expression of follicle-stimulating (10 and 100 mg/kg), androgen (100 mg/kg), estrogen (100 mg/kg), progesterone (all doses) and peroxisome proliferator-activated (all doses) receptors, 3β hydroxysteroid dehydrogenase (all doses), aromatase and steroidogenic acute regulatory proteins (all doses) in the ovary; accelerated rate of follicle recruitment (10 and 100 mg/kg)	[111]
Mouse	Female	DEHP from E11 until birth	20, 200 µg/kg/day 500, 750 mg/kg/day	F1: estrous cyclicity impairment (750 mg/kg); increased ovarian cysts number (750 mg/kg); total follicle number decrease (750 mg/kg); increased estradiol levels (500 and 750 mg/kg); decreased testosterone (500 mg/kg), inhibin B (750 mg/kg) and FSH levels (500 mg/kg); increased LH levels (20 µg/kg) F2: altered follicle numbers (200 µg/kg and 500 mg/kg); decreased testosterone (20 µg/kg); decreased progesterone (200 µg/kg) F3: estrous cyclicity impairment (20 and 200 µg/kg and 500 and 750 mg/kg); decreased follicle numbers (200 µg/kg/d and 500 mg/kg); increased estradiol levels (20 µg/kg); decreased testosterone (20 µg/kg and 500 mg/kg); decreased inhibin B levels (500 mg/kg); increased FSH (500 mg/kg) and LH levels (500 mg/kg)	[107]
Mouse	Female	DEHP from E10.5 until birth	20 and 200 μg/kg/day 500, and 750 mg/kg/day	F1: accelerated puberty onset (200 µg/kg); disrupted estrous cyclicity (200 µg and 500 mg/kg); altered folliculogenesis (20 and 200 µg/kg); increased Dnmt expression in the ovary (750 mg/kg); increased presence of 5-mC in the ovary (20 µg/kg). F2: accelerated puberty onset (500 mg/kg); disrupted estrous cyclicity (20 and 200 µg/kg); increased 17β-estradiol levels (20 μg/kg); decreased expression of steroidogenic enzymes in the ovary (20 μg/kg); dysregulation of PI3K factors in the ovary (20 and 200 µg/kg; 750 mg/kg); decreased Tet expression in the ovary (all doses). F3: accelerated puberty onset (20, 200 µg, and 500 mg/kg); disrupted estrous cyclicity (20 µg/kg/day); decreased expression of steroidogenic enzymes in the ovary; decreased Dnmt in the ovary (all doses) and Tet expression in the ovary (200 μg/kg; 500 and 750 mg/kg) and 5-mC levels in the ovary (500 and 750 mg/kg)	[85,112]
Mouse	Female	From E10 to birth	Mixture of 20 and 200 μg/kg/day—200 and 500 mg/kg/day [DEP (35.22%), DEHP (21.03%), DBP (14.91%), DiBP (8.61%), DiNP (15.10 %), and BzBP (5.13 %)]	F1: decreased FSH (500 mg/kg), estradiol (20 µg/kg; 200 and 500 mg/kg) testosterone (200 µg/kg; 200 and 500 mg/kg levels) and progesterone (500 mg/kg) levels; decreased steroidogenesis (20 and 200 µg/kg; 500 mg/kg); altered transition among follicle types (20 μg/kg and 200 mg/kg); higher incidence of atresia (500 mg/kg) F2 and F3: increased number of cystic ovaries (all doses); breeding, pregnancy and delivery complications (20 µg/kg and 500 mg/kg)	[106,107,108]
Mouse	Female	DEHP from E0.5 to PND ^+^ 21	0.05, 5 mg/kg/day	F1-F3: accelerated follicular recruitment (all doses); reduction of primordial follicular reserve (all doses); increased pre-antral follicles number (all doses); diminished oocyte quality (0.05 mg/kg); diminished embryonic developmental competence (0.05 mg/kg); altered expression profile of ovarian and pre-implantation embryonic genes, observed in the ovary and in blastocysts, respectively (all doses)	[113]

^+^ PND: postnatal day; * BW: body weight.

### 3.4. Pesticide

Several diffused endocrine disruptor pesticides have been shown to impact mammalian male and female reproductive health. Among these, atrazine, vinclozolin, methoxychlor (MTX), p,p′-dichlorodiphenoxydichloroethylene (DDE) and dichlorodiphenyltrichloroethane (DDT) [86] all exert both multi- and transgenerational inheritance (Table 4).

When gestating female rats were exposed to atrazine, a commonly used herbicide in the agricultural industry, from E8 to E14, their F1 generation male offspring had normal fertility (Table 3); instead, the F2 and F3 males showed an increased frequency of alterations of the seminiferous epithelium, mammary tumors and early puberty onset. In all generations, the sperm DMRs were identified, and specifically for the F3 generation, unique sets of epimutations were found to be associated with the lean phenotype or testes dysfunction [114] (Table 4).

The exposure to vinclozolin, another widespread pesticide, during the same time window induced increased spermatogenic cell apoptosis, decreased sperm motility and concentration and a drop in the epididymal sperm number in the F1–F3 rat generations [115]. The transgenerational actions of vinclozolin included an epigenetic reprogramming of the male germ line [116,117], which involved modification of the methylation level of several imprinted genes. Specifically, in the F1 and F2 offspring sperm, the number of methylated CpG sites on the *H19* and *Gtl2* imprinted genes was reduced, whereas that of *Peg1*, *Snrpn* and *Peg3* was increased. In the F3 offspring, the level of methylated CpG sites of *Gtl2*, *Peg1* and *Snrpn* was restored, whereas its difference in *H19* and *Peg3* was significantly less marked, showing a gradual disappearance of vinclozolin effects through the generations [118]. Following a genome-wide DMRs analysis, F1 vinclozolin-exposed rat males showed fewer and, for the most part, distinct modified DMRs in sperm compared to those of F3 [119]. The authors suggested that the presence of altered DMRs epimutations in F1 promotes modifications that lead to the appearance of diverse DMRs through the generations [119]. Similarly, Gillette and collaborators showed that, at a lower vinclozolin dose (Table 4), one-third of altered rat sperm DMRs overlap in F1 and F3 males [120]. In another recent paper, it was reported that, after vinclozolin exposure, F1 generation sperm had a low number of DMRs, the F2 an increased number and the F3 generation has the highest number of DMRs. The comparison of DMRs revealed a minimal overlap between the F2 and F3 generations, since the majority of the modifications in the sperm were unique. Additionally, on the same samples, the analysis of the ncRNA revealed that the F1 generation had a higher number than that of the F3 and that F3 had a lower number compared that of F2. Interestingly, each generation has unique ncRNAs, as well as DMRs, indicating that they were not invariantly inherited across F1–F3. In addition, F3 had an increased number of differential histone retention sites (DHRs) [121] (Table 4).

Comparable effects were also described in the mice, in which transgenerational adult-onset reproductive diseases were confirmed mainly in the F3 male generation, where prostate abnormalities were observed together with a loss of spermatogenic activity, reduction in germ cells and azoospermia. The analysis of the F3 generation sperm epigenome identified differential DMRs, indicating that the mechanism behind the vinclozolin-induced transgenerational inheritance is an epigenetic reprogramming of the male germ line that occurs during gonadal sex determination. It has been suggested that the maintenance of sperm epigenome alterations up to the third generation could be the result of the protection from demethylation, as it occurs in imprinted DNA methylation sites [117,122] (Table 4).

Similar data were obtained with DDT, the first pesticide developed and used from the beginning of the 1950s in agriculture and for the elimination of malaria, especially in North America, and banned in the early 1970s [123]. The exposure to this pesticide during the time of rat fetal gonadal development alters the expression of several noncoding RNAs and the DNA methylation sperm landscape in the F1–F3 generations. Additionally, the majority of the sperm DNA methylation changes were unique among the generations, and a significantly high number of new histone retention sites were found only in sperm of the F3 generation [124] (Table 4).

DDE, a breakdown product of DDT, is another diffused pesticide that displays antiandrogen activity. Pregnant rat females exposed during a critical window for testis development (E8–E15) generated F1 males that displayed infertility [125]. They showed abnormal testis histology, a decrease in motile sperm concentration and the sperm fertility index; these features also transmitted to the following F2 and F3 generations [110] (Song and Yang 2018). In the embryonic testes of DDE-exposed F1 and F2 animals, Dnmt 1 and 3a were significantly downregulated, returning to the control levels only in the F3 male generation. However, DMR2 hypomethylation of the *Igf2* [125], *H19* and *Gtl2* [126] imprinted genes was reported up to F3 animals [125,126] (Table 4).

Pesticides also impair female reproductive health across generations. Exposure to atrazine during rat gonadal sex determination led to an F2 female generation with an increased frequency of mammary tumors [114]. In the mice, exposure to vinclozolin at the same phase of development promoted polycystic ovarian disease in F3 females, with very large cystic structures [122]. Additionally, granulosa cells of 20-day-old rat females of the F3 generation showed 164 and 293 differentially methylated regions after ancestral vinclozolin or DDT exposure, respectively, as well as several differentially expressed coding and noncoding RNAs in both pesticide-exposed lineages [127] (Table 4). Exposure to these pesticides during the early developmental phases induced modifications that predispose them to the development of ovarian diseases (i.e., polycystic ovarian syndrome and primary ovarian insufficiency) that were observed in the not-directly exposed F3 generation.

**Table 4:** Pesticide-induced effects in model animals across generations.

Species	Sex	Exposure	Dose	Effects across Generations	Reference
Rat	Male	Atrazine from E8 to E14	25 mg/kg BW/day	F2 and F3: azoospermia; atretic seminiferous tubules; vacuoles in the basal region of seminiferous tubules; sloughed germ cells; lack of seminiferous tubule lumen; high frequency of spermatogonia apoptosis; mammary tumors; early onset puberty; epimutations in spermatozoa F1–F3: epimutations in spermatozoa	[114]
Rat	Male	Vinclozolin from E8 to E14	100 mg/kg/ day	F1: lowest number of DMRs in spermatozoa; altered quantity of lncRNA in spermatozoa F2: increased number of DMRs in spermatozoa; altered quantity of lncRNA in spermatozoa F3: the highest number of DMRs in spermatozoa; altered quantity of lncRNA in spermatozoa; increased number of differential histone retention sites (DHRs) in spermatozoa	[121]
Mouse/Rat	Male	Vinclozolin from E8 to E14	100 mg/kg/ day (rat) 50 mg/kg/day (mouse) 100 mg/kg BW/day (rat; [115]) 1 mg/kg/day (rat; [120])	F1–F3: increased spermatogenic cell apoptosis; decreased sperm number and motility; drop of epididymal sperm number; epigenetic alterations in spermatozoa (DMRs modified) (all doses)	[115,116,117,118,119,120]
Rat	Male	DDT from E8 to E14	25 mg/kg BW/day	F1–F3: altered DNA methylation; altered noncoding RNAs expression in spermatozoa	[124]
Rat	Male	DDE from E8 to E15	100 mg/kg BW/day	F1 and F2: downregulation of DNMT1 and DNMT3 in the testis F1–F3: infertility; decreased motile sperm concentration; decreased sperm fertility index; altered testis morphology; altered imprinted gene expression in spermatozoa	[125,126]
Mouse	Female	Vinclozolin from E7 to E13	50 mg/kg BW/day	F3: polycystic ovary	[122]
Rat	Female	Vinclozolin or DDT from E8 to E14	100 mg/kg BW/day (Vinclozolin) 25 mg/kg BW/day (DDT)	F3: differentially methylated regions in granulosa cells; altered expression of RNAs (492 sncRNAs and 123 lncRNAs in the vinclozolin-exposed granulosa cells; 1085 sncRNAs and 51 lncRNAs in the DDT granulosa cells; 174 mRNAs in vinclozolin-exposed granulosa cells; 212 mRNAs in DDT-exposed granulosa cells; predisposition to ovarian diseases)	[127]

BW: body weight.

### 3.5. Persistent Environmental Contaminants

Some EDCs persist in the environment for long periods of time, even after their banning, as a consequence of insolubility, lipophilicity and high resistance to degradation. This category of EDCs includes polychlorinated biphenyls (PCBs) and several dioxins (e.g., TCDD) known to affect both male and female fertility [128]. Despite their importance for the environment, only a few studies, reported below, have addressed their multi- and transgenerational effects on reproduction (Table 5).

Ancestral exposure to TCDD from E8 to E14 induced several epimutations in the genes involved in ribosome and chemokine signaling pathways, natural killer cell-mediated toxicity in sperm and a drop in the testosterone levels in the male F3 rat generation [96]. Following ancestral exposure to Aroclor 1221, a mixture of polychlorobiphenyls, from E8 to 18, the number of DMRs in the rat F1 sperm was twice compared to that found in the F3 [120] (Table 5).

When mouse embryos were exposed, during the whole in utero developmental phase and then, after birth, up to PND21, to a mixture of two PCBs congeners, the F1–F3 male progeny showed reduced testis weight, seminiferous tubule diameters and sperm viability [129]. Longer exposure (5 weeks before mating and, following fertilization, up to delivery) to a mixture of PCBs and of organochlorine pesticides induced altered prostate weight, testosterone level, puberty, lower sperm quality and subfertility in the F1 and F2 generations of males. This complex reproductive phenotype was restored in F3, suggesting that these persistent environmental contaminant-associated impairments emerge in directly exposed F1 and F2 males but do not pass on to not-directly exposed animals. At the molecular level, the DNA methylome analysis of epididymal sperm highlighted a slightly greater similarity between F1 and F2 but was overall comparable among the three generations, although *Dnmt3l*, an important coactivator of Dnmt3a and b *de novo* methyltransferases, was found hypermethylated in F1 but hypomethylated in F2 and F3 [130] (Table 5).

The effects of persistent environmental contaminants are also reported in females. Specifically, exposure to TCDD during rat fetal gonadal sex determination induced primordial follicle loss and increased the probability of developing polycystic ovary disease in 1-year-old F1 females [96]. In mice, exposure to TCDD on E15.5 induced adenomyosis and reduced fertility, dysmenorrhea [131] and preterm birth [132] in the female F3 generation. Additionally, exposure during fetus development from E16 to E18 to a mixture of PCBs (A1221) generated healthy F1 offspring, but starting from F2, the females showed altered serum progesterone (lower in F2 and higher in F3) and higher serum estradiol (in F3) concentrations, impacting their fertility [133]. Prolonged exposure from E0 to PND21 resulted in reduced F1 ovary weight, a lower oocyte developmental capacity and increased follicular atresia [129] (Table 5).

**Table 5:** Persistent environmental contaminant-induced effects in model animals across generations.

Species	Sex	Exposure	Dose	Effects across Generations	Reference
Rat	Male	TCDD from E8 to E14	100 ng/kg BW */day	F3: sperm epigenome alteration; reduction of testosterone levels	[96]
Rat	Male	A1221 (mixture of PCBs) from E8 to E18	1 mg/kg BW/day	F1 and F3: epigenetic alterations in spermatozoa (DMRs modified)	[120]
Rat	Male	POP mixture (polychlorinated biphenyls and organochlorine pesticides)	500 µg/kg BW three times a week for 5 weeks, before mating through mating and parturition of the F1 litters	F1: decreased conception; decreased fertility; reduced number of fetuses; low sperm quality; advanced puberty; lower testosterone concentration; small epididymis; low prostate weights; reduced sperm counts; reduced sperm motility; hyper-methylation of *Dnmt3l* gene in spermatozoa F2: decreased fertility reduced number of fetuses; low sperm quality; delayed puberty; lower testosterone concentration; small epididymis; low prostate weights; reduced sperm counts; hypo-methylation of *Dnmt3l* gene in spermatozoa F3: hypo-methylation of *Dnmt3l* gene in spermatozoa	[130]
Mouse	Male	PCBs (mixture of two congeners) from E0 to PND ^+^ 21	0, 1, 10, and 100 µg PCB/kg BW/day	F1 and F2: reduced testis weight (all doses); reduced seminiferous tubule diameter (all doses); low sperm viability (all doses); reduced fertility (all doses)	[129]
Rat	Female	TCDD from E8 to E14	100 ng/kg BW/day	F1: primordial follicle loss; polycystic ovary disease	[96]
Mouse	Female	TCDD on E15.5	10 µg/kg BW/day	F3: adenomyosis; reduced fertility; dysmenorrhea; preterm birth	[131,132]
Rat	Female	A1221 from E16 to E18	1 mg/kg BW/day	F2 and F3: altered serum progesterone and estradiol levels; low fertility	[133]
Mouse	Female	PCBs (mixture of two congeners) from E0 to PND 21	0, 1, 10, and 100 µg/kg BW/day	F1: reduced ovary weight (all doses); low oocyte developmental capacity (100 µg/kg); increased follicular atresia (all doses); smaller litters (all doses)	[129]

^+^ PND: postnatal day; * BW: body weight.

## 4. Epigenetic Mechanisms of Transmission across Generations

**Figure 2:**
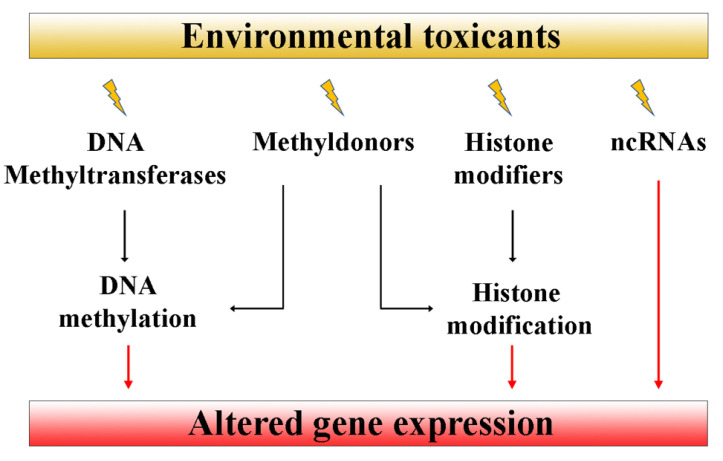
EDC exposure may alter DNA methyltransferases, histone modifiers and noncoding RNAs with downstream effects on the gene expression.

Altered reproductive phenotypes pass across generations through the germline inheritance of EDC-induced epigenetic changes [26,31]. A consistent number of papers have proposed that changes in DNA methylation, in the post-translational modification of histone tails and in the expression of noncoding RNAs are likely involved in a nongenetic transfer of environmental information [26,121,124,127,130,134,135,136]. However, the exact molecular mechanisms by which EDCs alter epigenetic marks remain, to date, still unknown. It has been suggested that these compounds may affect the abundance and/or the activity of some epigenetic regulators, e.g., ncRNAs, histone modifiers and DNA methyltransferases, and/or of their cofactors, e.g., methyl donors, which, in turn, act by modifying the gene expression (Figure 2).

In rat F1 testicular Leydig cells, prenatal exposure to DEHP phthalate at 1, 10 and 100 mg/kg/day during E9–21, the RNA and protein expressions of *Dnmt1* (maintenance methyltransferase) and *Dnmt3A/Dnmt3B* (de novo methyltransferases) [137,138] were upregulated [134]. This upregulation was associated with a decreased gene expression of steroidogenic factor-1 and specific protein-1 transcription factors, whose promoters were found hypermethylated [134]. Hypermethylation of the *Dnmt3l* gene, an important coactivator of Dnmt3a and b activity, was found in the F1 rat sperm generation, ancestrally exposed to a mixture of polychlorinated biphenyls and organochlorine pesticides, but not in F2 and F3, which, instead, were hypomethylated [130]. The authors suggested that direct exposure to long, persistent environmental contaminants can modify the methylation of key methylation enzyme genes, which might be restored in subsequent generations [130].

In the mice ovaries, following prenatal exposure to DEHP from E10.5 until birth, the expression of DNA methyltransferases and Tet enzymes varied at different doses and among the F1–F3 generations. In F1, at the highest dose (Table 3), the mRNA *Dnmt1* level was increased, parallel to an increased percentage of 5-mC [26]. In F2, while the *Dnmt1* expression returned to the control values, *Tet1* (an enzyme primarily responsible for oxidizing 5-mC into 5-hydroxymethyl cytosine (5-hmC) [139]), as well as the *Tet2* and *Tet3* (which oxidize 5-hmC into further oxidized cytosines that are replaced with an unmethylated, unmodified cytosine [140,141]) levels, were downregulated at different doses. This downregulation suggests that the DNA demethylation pathways are affected but not to a degree enough to significantly decrease the 5-mC percentage, whose level was unaltered. In the F3 generation, the expression of *Dnmt1, Dnmt3a*, *Dnmt3b*, *Tet2* and *Tet3*, but not of *Tet1* [26], was downregulated at different doses, as it was the percentage of 5-mC [26]. Whether the variation in the 5-mC level may contribute to altering the methylation of the genes involved in critical ovarian functions that can be passed down the generations is still to be determined.

Another mechanism through which EDCs may exert their multi- or transgenerational effects on reproduction is the post-translational modifications of histones, e.g., methylation, acetylation, phosphorylation, ubiquitination, biotinylation, sumoylation and ADP-ribosylation. How EDCs affect histones and/or their modifications is almost unclear, and to date, it has yet to be proven how these alterations could be transmitted from one generation to the next [136]. We know that EDCs have, as a major target, the nuclear hormone receptors, which regulate the transcription of specific target genes, and they require coactivators, some of which possess histone acetyltransferase (HAT) activity [142]. In this regard, in humans, it has been shown that HAT activity is induced following tributyltin or triphenyltin, two synthetic EDCs [143]. Histone retention in sperm chromatin could be another mechanism of epigenetic transgenerational inheritance. For example, histone H3K27me3 retention has been observed in F3 rat sperm exposed to 25 mg/kg BW/day DTT and 100 mg/kg BW/day vinclozolin during E8–14 [121,124].

Noncoding RNAs, molecules known to be involved in gene expression regulation, were proposed to play a significant role in carrying epigenetic information across generations. Environmental contaminants can affect the production of ncRNAs, especially miRNAs, lncRNAs and piRNAs. In the mice PGCs, vinclozolin exposure during the entire pregnant period prompted a decrease of mmu-miR-23b and mmu-miR-21 miRNAs, which, in turn, downregulated the Lin28/let-7/Blimp1 PGC specification pathway in the three successive generations [135]. Prenatal exposure to vinclozolin or to DTT induced, in F3 rat granulosa cells, a transgenerational differential expression of miRNAs and lncRNAs [136]. In sperm, DTT prenatal exposure induced an increasing number of differentially expressed ncRNA in F1–F3, and piRNAs were the most abundant in the three generations [124]. It has been recently suggested that environmental-induced DNA methylation may impact adjacent ncRNA genes, modifying their expression and thus affecting their target gene expression. Noncoding RNA production may also be directly affected by environmental agents, eventually leading to altered DNA methylation patterns and, consequently, gene expression [9].

## 5. Discussion and Conclusions

ET-induced heritable alterations negatively impact on reproductive functions representing a biomedical and environmental key issue for human society that need to be addressed by both intensive biomedical and environmental research. Controlled laboratory studies performed on model animals exposed during the fetal or the early postnatal periods clearly showed the impact of ETs on mammalian male and female gametogenesis, fertility and reproductive health. In the males, they alter the correct spermatogenic process, the maturation of germ cells, induce apoptosis and azoospermia and decrease sperm motility. In the females, they mainly impair the correct progression of follicle maturation, induce follicle atresia and modify estrous cyclicity. These alterations and the severity of the phenotype are not always directly related to the compound or to its exposure dose, suggesting the absence of a monotonic dose–response relationship. This feature, typical of several ETs, complicates the correct understanding of the biochemical pathways and the molecular and cellular mechanisms of the damages elicited by the compounds, but also, it makes it difficult to define reliable environmental tolerance thresholds. When the exposure occurs in utero, the ET detrimental effects induced on the F1 generation may be transmitted to the next generations, even at F3, which is not directly exposed, with a severity that may be dependent on the compound. These multi- and transgenerational inheritances may be mediated by epigenetic alterations, such as DNA methylation manifested as DMRs located throughout the genome, which, in turn, may affect the expression of multiple genes. ET-induced differential DMR levels in both male and female germ cells might be related to the altered expression of Dnmt or Tet enzymes. Other epigenetic mechanisms are likely involved in the inheritance across generations, such as histone post-translational modifications and variations in ncRNA expression, but the knowledge of their involvement in environmental-mediated inheritance is very limited.

Another aspect that remains scarcely known is concerned with the effects exerted by ETs on the reproduction of wild mammalian populations, which might have negative impacts on the maintenance of the species. To have a comprehensive understanding of ET actions on reproduction, research should be performed in parallel at two different levels: (1) in traditional controlled laboratory conditions and (2) extending the studies to their natural context to better understand how natural populations with their range of genetic variability mediate physiological responses to their very complex mixture and concentration of compounds at the individual and population levels and across ecological systems.

Lastly, as toxicant exposure is a reality that humans experience worldwide and that, at present, cannot be eluded, long-term follow-up studies in humans are needed to further investigate the association between exposure and the risk of reproductive dysfunctions throughout generations and for planning public health policies.

## Data Availability

Not applicable.
